# Determinants and Gaps in Preventive Care Delivery for Indigenous Australians: A Cross-sectional Analysis

**DOI:** 10.3389/fpubh.2016.00034

**Published:** 2016-03-10

**Authors:** Christopher Bailie, Veronica Matthews, Jodie Bailie, Paul Burgess, Kerry Copley, Catherine Kennedy, Liz Moore, Sarah Larkins, Sandra Thompson, Ross Stewart Bailie

**Affiliations:** ^1^School of Medicine, University of Queensland, Brisbane, QLD, Australia; ^2^Menzies School of Health Research, Charles Darwin University, Darwin, NT, Australia; ^3^Primary Health Care Branch, Top End Health Service, Northern Territory Government, Darwin, NT, Australia; ^4^Aboriginal Medical Services Alliance NT (AMSANT), Darwin, NT, Australia; ^5^Maari Ma Health Aboriginal Corporation, Broken Hill, NSW, Australia; ^6^College of Medicine and Dentistry, James Cook University, Townsville, QLD, Australia; ^7^Anton Breinl Research Centre for Health Systems Strengthening, Australian Institute of Tropical Health and Medicine, James Cook University, Townsville, QLD, Australia; ^8^Western Australian Centre for Rural Health, School of Primary, Aboriginal and Rural Health Care, University of Western Australia, Geraldton, WA, Australia; ^9^School of Population Health, University of Queensland, Brisbane, QLD, Australia

**Keywords:** preventive healthcare, Aboriginal and Torres Strait Islander, variation, indigenous, quality of care, adherence to best practice guidelines

## Abstract

**Background:**

Potentially preventable chronic diseases are the greatest contributor to the health gap between Aboriginal and Torres Strait Islander peoples and non-­Indigenous Australians. Preventive care is important for earlier detection and control of chronic disease, and a number of recent policy initiatives have aimed to enhance delivery of preventive care. We examined documented delivery of recommended preventive services for Indigenous peoples across Australia and investigated the influence of health center and client level factors on adherence to best practice guidelines.

**Methods:**

Clinical audit data from 2012 to 2014 for 3,623 well adult clients (aged 15–54) of 101 health centers from four Australian states and territories were analyzed to determine adherence to delivery of 26 recommended preventive services classified into five different modes of care on the basis of the way in which they are delivered (e.g., basic measurement; laboratory tests and imaging; assessment and brief interventions, eye, ear, and oral checks; follow-up of abnormal findings). Summary statistics were used to describe the delivery of each service item across jurisdictions. Multilevel regression models were used to quantify the variation in service delivery attributable to health center and client level factors and to identify factors associated with higher quality care.

**Results:**

Delivery of recommended preventive care varied widely between service items, with good delivery of most basic measurements but poor follow-up of abnormal findings. Health center characteristics were associated with most variation. Higher quality care was associated with Northern Territory location, urban services, and smaller service population size. Client factors associated with higher quality care included age between 25 and 34 years, female sex, and more regular attendance.

**Conclusion:**

Wide variation in documented preventive care delivery, poor follow-up of abnormal findings, and system factors that influence quality of care should be addressed through continuous quality improvement approaches that engage stakeholders at multiple levels (including, for example, access to care in the community, appropriate decision support for practitioners, and financial incentives and context appropriate guidelines).

## Introduction

Worldwide, Indigenous populations experience poorer health than their non-Indigenous counterparts (1). The greatest contributor to the gap in health outcomes between Aboriginal and Torres Strait Islander^1^ peoples and the general Australian population is potentially preventable chronic disease (2). The critical role of preventive healthcare in curbing the impact of chronic disease is now widely recognized (3, 4). However, reported levels of delivery of recommended preventive care to Indigenous Australians still have much room for improvement (5–9).

Australians have access to Medicare, a universal health insurance scheme. Indigenous Australians access primary health care (PHC) through both private general practice and primary health services designed to meet the needs of Indigenous ­peoples. These include Aboriginal community-controlled health ­services (ACCHS) and government operated Indigenous-specific services (10).

Recently, a number of Australian Government policy initiatives have attempted to improve prevention for Indigenous Australians, including the Indigenous Chronic Disease Package (ICDP) from 2009 to 2013 (11). Preventive care priorities within the ICDP were: (a) smoking cessation; (b) increasing uptake of preventive health assessments and follow-up of abnormal findings; (c) workforce training to improve access to care and preventive health care delivery.

Preventive care for Indigenous Australians is incentivized by the Medicare Benefit Schedule (MBS) 715 annual “Health Assessment for Aboriginal and Torres Strait Islander People” that has achieved better identification of chronic disease and its risk factors as well as reduction in cardiovascular disease risk (12–14). Continuous quality improvement (CQI) in Indigenous PHC services has proved effective in improving preventive care delivery through enabling PHC services to identify and address barriers to preventive care (15).

The Audit and Best Practice for Chronic Disease National Research Partnership (ABCD NRP) and One21seventy have ­supported the use of CQI in Indigenous PHC by providing evidence-based clinical audit and systems assessment tools and through training and assistance with their use (16, 17). More than 270 health centers across Australia have used ABCD/One21seventy tools and processes to improve their quality of care (18, 19). Data collected from over 170 health centers are available for research purposes through the ABCD NRP.

Obtaining reliable data on delivery of care in relation to best practice guidelines are critical in targeting strategies to improve performance (6). Data to date for individual preventive service items have shown wide variation in delivery between clients and health centers (5–8, 14, 20).

Overall measurement of service delivery may lack the detail needed to identify specific opportunities for improvement in quality of care (21). Understanding performance in relation to the mechanisms by which care processes are delivered (referred to as “modes of care”), for example: basic measurements, lifestyle interventions, laboratory tests, and so on, may allow better insight into higher level system changes needed to improve care quality (21).

The aim of this paper is to assess variation in delivery of preventive health care to Indigenous people using data collected through the ABCD NRP. Specifically, our objectives are to: (1) assess differences in delivery of recommended preventive services; (2) examine health center and client level factors associated with quality care; (3) examine how these factors vary across different modes of care.

## Materials and Methods

### Setting

ACCHS and government operated Indigenous health centers participating in the ABCD NRP were included in this study. These health centers vary in size from small services with 1–2 nursing staff to large services with a range of medical, nursing, and allied health professionals. They predominantly but not exclusively serve Indigenous clients.

### Data Sources

As part of their routine CQI activities, participating health centers performed annual audits of client medical records to determine whether recommended preventive service items were documented as delivered in the previous 24 months. Audit inclusion criteria were: (1) age between 15 and 55 years; (2) resident in the community for at least six of the last 12 months; (3) no diagnosis of diabetes, hypertension, coronary heart disease, chronic heart failure, rheumatic heart disease or chronic kidney disease; (4) not pregnant or not less than 6 weeks postpartum at the time of audit; (5) attendance at the health center in the previous 24 months.

Health centers were encouraged to audit all client records if their eligible client population was less than 30. For eligible client populations greater than 30, health centers audited a random sample of records of at least 30 eligible clients.

Samples were stratified by age and gender. Data were available from 101 health centers spread across four Australian states and territories over the period 2012–2014. Data from the most recent preventive services clinical audit from each health center were included in our analysis.

### Measures

The One21seventy/ABCD NRP preventive services audit tool contains 26 items recommended for delivery at least every 24 months. We classified items into five modes of care: basic measurements; laboratory and imaging; eye, ear and oral checks; assessment and counseling for lifestyle risk factors; and follow-up of abnormal findings (Table 1). A service was recorded as delivered for each eligible client if there was clear record of delivery at least once within the previous 24 months.

**Table 1:** **Percentage delivery of preventive service items by jurisdiction**.

		**QLD**	**SA/WA**	**NT**	**Total**
	
	No. of client records	1,561	342	1,720	3,623
	No. of health centers	45	8	48	101
	
**Service item**	**Relevant population for service item**	**% delivery (standard error of the mean)**			

**Basic measurements**					
Weight*	Well adults 15–54	61.9 (1.2)	82.5 (2.1)	81.6 (0.9)	73.2 (0.7)
Body mass index*		26.8 (1.1)	70.8 (2.5)	58.2 (1.2)	45.9 (0.8)
Waist circumference*		18.6 (1.0)	36.3 (2.6)	55.2 (1.2)	37.6 (0.8)
Blood pressure^†^		80.3 (1.0)	84.5 (2.0)	88.8 (0.8)	84.7 (0.6)
Pulse rate^†^		68.2 (1.2)	67.8 (2.5)	85.6 (0.8)	76.4 (0.7)
Urinalysis^†^		34.5 (1.2)	16.4 (2.0)	64.5 (1.2)	47.0 (0.8)
Blood glucose level^†^		54.3 (1.3)	72.2 (2.4)	75.6 (1.0)	66.1 (0.8)
**Laboratory and imaging investigations**					
NAAT for gonorrhea and chlamydia^†^	Well adults 15–34 years sexually active^a^	55.0 (1.6)	25.8 (2.9)	73.3 (1.3)	61.5 (1.0)
Syphilis serology^†^		51.4 (1.6)	10.0 (2.0)	54.8 (1.4)	49.4 (1.0)
Serum lipids*	Well adults ≥35; or 18–34 with either obesity, smoker, elevated BP, or family history of premature CHD or CKD^b^	27.9 (1.3)	20.2 (2.5)	69.2 (1.3)	46.2 (0.9)
Pap smear*	Well females 18–54 years who have been sexually active^a^	50.2 (1.9)	38.6 (4.2)	54.5 (1.8)	51.3 (1.2)
Mammography*	Well females 50–54 years at average risk of breast cancer, younger if increased risk^c^	19.6 (5.9)	0.0 (0)	20.0 (6.0)	17.6 (3.8)
**Assessment and brief intervention for lifestyle risk factors**					
Smoking status recorded*	Well adults 15–54 years	52.4 (1.3)	79.5 (2.2)	64.1 (1.2)	60.5 (0.8)
Alcohol use status recorded*		49.3 (1.3)	74.9 (2.3)	58.7 (1.2)	56.2 (0.8)
Brief intervention for smoking*	Current smokers	59.9 (2.3)	61.9 (3.7)	73.1 (1.7)	66.8 (1.3)
Brief intervention for alcohol use*	Hazardous or harmful alcohol use	71.6 (3.7)	50.7 (5.8)	79.0 (2.7)	71.9 (2.1)
Brief intervention for overweight/obese*	High BMI or waist circumference	54.4 (3.0)	18.0 (3.1)	48.4 (2.0)	45.8 (1.5)
Reproductive and sexual health discussion*	Well adults 15–54 years	49.4 (1.3)	41.8 (2.7)	55.6 (1.2)	51.6 (0.8)
**Eye, ear and oral checks**					
Oral Health Check*	Well adults 15–54 years	33.6 (1.2)	47.7 (2.7)	54.7 (1.2)	44.9 (0.8)
Ears & Hearing Assessment*		31.8 (1.2)	47.7 (2.7)	55.9 (1.2)	44.7 (0.8)
Visual acuity*	Well adults ≥40 years	28.3 (2.3)	32.9 (5.1)	38.1 (2.6)	33.0 (1.6)
Eye assessment for Trichiasis*	Well adults ≥35 years in trachoma endemic areas^d^	1.2 (0.5)	28.6 (17.1)	35.0 (2.2)	17.6 (1.2)
Composite indicator		48.1 (0.8)	55.3 (1.3)	67.9 (0.8)	58.2 (0.5)
**Follow-up of abnormal findings**					
Follow-up for abnormal serum lipid profile^‡^	Adults with abnormal lipid profile	27.5 (2.6)	37.2 (7.4)	23.6 (1.5)	25.1 (1.3)
Follow-up for abnormal blood pressure measurement^‡^	Adults with abnormal BP	31.2 (4.4)	20.0 (5.7)	27.7 (3.9)	27.7 (2.6)
Follow-up for abnormal blood glucose measurement^‡^	Adults with abnormal glucose tests	18.1 (2.2)	6.5 (2.2)	17.7 (1.6)	16.5 (1.2)
Follow-up for protein on urinalysis^‡^	Adults with 1+ or more protein on urinalysis	61.3 (4.7)	80.0 (12.7)	59.9 (3.6)	61.1 (2.8)

*Number of client records and health centers are overall for each jurisdiction. The actual number of client records is lower for some service items that are recommended for restricted populations*.

*To calculate delivery we assumed that: ^a^all adults had been sexually active; ^b^that adults had no family history of premature CHD or CKD; ^c^that females were not at above average risk of breast cancer; for ^d^that all remote locations were trachoma endemic areas*.

*NAAT, nucleic acid amplification test; BP, blood pressure; CHD, chronic heart disease; CKD, chronic kidney disease*.

*National guide to a preventive health assessment for Aboriginal and Torres Strait Islander people (22).

*^†^Standard treatment manual (23)*.

*^‡^Chronic disease guidelines (24)*.

Four follow-up items were included in the audit. These were follow-up for high blood pressure (BP), for protein on urinalysis, for high blood glucose level (BGL), and for abnormal lipid profile. Definitions of adequate follow-up, respectively, were a documented plan for: (1) repeat BP measurement in 2–4 weeks; (2) testing albumin-creatinine ratio; (3) re-testing BGL; (4) ­re-testing lipid profile. Denominators for calculating indicators of follow-up were based on numbers of clients who had an abnormal result for the relevant clinical/laboratory investigation within the past 2 years.

Some guidelines restrict recommended delivery of some services to special populations, for example, based upon a family history or when a person is sexually active. In cases where audit data were insufficient to define these special populations we made the following assumptions in order to estimate delivery: (a) for items related to sexual and reproductive health, we assumed that all young adults had been sexually active [noting the high rate of sexual activity among young Australians (25)]; (b) for serum lipid testing, we assumed that no clients had a family history of premature coronary heart disease or chronic kidney disease; (c) for mammography, we assumed that no female clients were at above average risk of breast cancer; (d) for trachoma screening, we assumed that all remote health centers were located in trachoma endemic areas, and therefore that screening was recommended [noting the higher rates of trachoma in remote and very remote areas (26)].

Health center characteristics included, size of population served, governance (ACCHS or government operated), and location (urban, regional, or remote). Duration of CQI participation has been shown to be associated with improved quality of care (27) and was therefore included in the analysis to correct for potential bias associated with different health centers participating in CQI for different lengths of time. The clinical audit data included information on the following client characteristics; age, gender, Indigenous status, and whether or not the client had attended the health center in the previous 6 months.

### Statistical Analysis

We used STATA version 13 software for statistical analysis. Using summary statistics, we described the mean delivery of each service item for each jurisdiction (Table 1).

For the purposes of the analysis, the data from South Australia (SA) and Western Australia (WA) health centers were pooled because (a) there were relatively small number of participating health centers (two from WA, six from SA); (b) service delivery levels were relatively similar for SA and WA compared to other jurisdictions; and (c) SA and WA had relatively less developed support structures for CQI processes compared to the Northern Territory (NT) and Queensland (QLD) over the period covered by this study.

Adherence to delivery of recommended service items was calculated for each mode of care by dividing the documented service items for that client by the total number of recommended service items in that mode of care. We also calculated adherence to delivery for an overall composite indicator that included all service items except those related to follow-up of abnormal findings (Table 1).

Aggregate scores for the composite indicator and each mode of care were converted into binary outcome indicators that categorized “higher” performance as being above the median (top 50%) of delivery for all clients across all health centers. “Lower performance” was categorized as being below or equal to the median.

We used multi-level mixed effects logistic regression models to quantify the variation in service delivery separately for the overall indicator and each mode of care. These models allowed for the hierarchical structure of the data (clients nested within health centers).

For each indicator, we calculated unadjusted odds ratios to measure the association between “higher” levels of delivery and each health center and client characteristic.

For the adjusted analysis, we used a stepwise modeling strategy starting with an “empty” model with no explanatory variables (Model A). Health center (Model B) and then client level variables (Model C) were then introduced into the empty model. Potential interactions were introduced into the final model (Model C) in a stepwise manner and their significance was tested. No interactions were found to make a meaningful difference, and none were included in the final analysis. The model was tested for sensitivity to alternate specifications (including alternative cut points for the outcome variable) during the model building process and was found to be generally robust.

The reduction in variance due to the stepwise introduction of the client and health center level variables in the models was determined by the proportional change in variance (PCV). The PCV provides an estimate of the extent to which these factors may explain differences in propensity for better delivery of health care (28).

We calculated median odds ratios (MORs) to interpret variance in the odds ratio scale. In the odds ratio scale, the MOR describes the increase in median probability of better delivery if a client was to move from one randomly picked health center to another (29). For a MOR equal to 1, there is no difference between health centers in their probability of adhering to the recommended service delivery. The greater the MOR, the greater the unexplained variability between health centers.

### Ethics Approvals

Ethics approval has been obtained for the ABCD NRP project research ethics committees in relevant jurisdictions of Australia. These include the Human Research Ethics Committee (HREC) of the Northern Territory Department of Health and Menzies School of Health Research (HREC-EC00153); Central Australian HREC (HREC-12-53); Queensland HREC Darling Downs Health Services District (HREC/11/QTDD/47); South Australian Aboriginal Health Research Ethics Committee (04-10-319); Curtin University HREC (HR140/2008); Western Australian Country Health Services Research Ethics Committee (2011/27); Western Australia Aboriginal Health Information and Ethics Committee (111-8/05); and University of Western Australia HREC (RA/4/1/5051).

## Results

Of the 101 participating health centers, 93 were located in the NT and QLD. More than 90% from QLD and the NT were located in a regional or remote area compared to 50% for SA and WA (Table 2). Fifty percent of health centers had a population of fewer than 500 people, 84% were government operated.

**Table 2:** **Health center and client characteristics by jurisdiction (*N* and % of total)**.

		**QLD**	**SA/WA**	**NT**	**Total**
		
**Health center level**	**No. of health centers audited**	**45**	**8**	**48**	**101**

Location	Urban	3 (7)	4 (50)	1 (2)	8 (8)
	Regional	5 (11)	3 (38)	2 (4)	10 (10)
	Remote	37 (82)	1 (13)	45 (94)	83 (82)
Population size	<500	23 (51)	2 (25)	26 (54)	51 (50)
	500–1,000	10 (22)	3 (38)	8 (17)	21 (21)
	>1,000	12 (27)	3 (38)	14 (29)	29 (29)
Governance	Community-controlled	1 (2)	4 (50)	11 (23)	16 (16)
	Government	44 (98)	4 (50)	37 (77)	85 (84)
CQI experience	Baseline audit	5 (11)	3 (38)	10 (21)	18 (18)
	1–2 follow-up audits	18 (40)	4 (50)	13 (27)	35 (35)
	≥3 follow-up audits	22 (49)	1 (13)	25 (52)	48 (48)

**Client level**	**No. of client records audited**	**1,561**	**342**	**1,720**	**3,623**

Gender of client	Male	772 (49)	186 (54)	858 (50)	1,816 (50)
	Female	789 (51)	156 (46)	862 (50)	1,807 (50)
Age group	15–24 years	625 (40)	129 (38)	666 (39)	1,420 (39)
	25–34 years	382 (24)	92 (27)	553 (32)	1,027 (28)
	35–44 years	324 (21)	62 (18)	294 (17)	680 (19)
	45–54 years	230 (15)	59 (17)	207 (12)	496 (14)
Indigenous status	Indigenous	1,265 (81)	316 (92)	1,666 (97)	3,247 (90)
	Non-Indigenous	166 (11)	26 (8)	46 (3)	238 (7)
	Not recorded	130 (8)	0 (0)	8 (0)	138 (4)
Time since last attendance	<6 months	1,067 (68)	191 (56)	1,413 (82)	2,671 (74)
	≥6 months	494 (32)	151 (44)	307 (18)	952 (26)

Records of 3,623 clients from these health centers were audited from February 2012 to December 2014. The sample size for each health center varied between 7 and 68 client records. Of these, 50% were females and 90% were Indigenous (Table 2).

The proportion of eligible clients receiving recommended care ranged from 18 to 85% for individual preventive services (Table 1). The mean delivery of the composite indicator including all service items except follow-up was 58%. Most basic measurements were delivered at levels of approximately 70–80% (Table 1); although recording of body mass index (BMI), waist circumference, and urinalysis were relatively low (37–47%). Most recommended laboratory and imaging investigations were delivered to approximately 50% of clients, apart from mammography which was delivered to 18% of eligible females. Eyes, ears, and oral checks were recorded as delivered to 18–45% of eligible clients. Delivery of service items to do with assessment and brief intervention for lifestyle risk factors ranged from 46 to 72%. Follow-up of abnormal findings was relatively low (17–28%) except for follow-up of positive protein on urinalysis (61%).

The unadjusted logistic regression analysis for the composite overall indicator showed significant effects for all factors except governance (Table 3). The health center MOR for the empty model for the overall indicator was 4.02 (Table 4; Model A), meaning if a client were to move from one randomly picked health center to another with higher delivery, they would have a 4.02 times higher chance (in median) of higher delivery. For the adjusted analysis for the overall composite indicator, the reduction in health center level variance for the addition of health center factors (PCV) was 60% (Table 4; Model B). Health center factors associated with higher levels of delivery included urban location, smaller service population, and location in the NT. Client level factors associated with higher delivery included being aged 25–34 years compared to other age groups, female gender, and more recent health center attendance (Table 4; Model C).

**Table 3:** Unadjusted multilevel logistic regression analyses of health center and client level factors on delivery of guideline-scheduled service items (*p < 0.05; **p < 0.01).

		**Overall**	**Basic measurements**	**Laboratory and imaging investigations**	**Eye, ear, and oral checks**	**Assessment and brief intervention for lifestyle risk factors**	**Follow-up of abnormal findings**
	
	No. of client records	3,623	3,623	3,623	3,623	3,623	1,905
	No. of health centers	101	101	101	101	101	101

		Odds ratio (95% confidence interval)					
**Health center level characteristics**							
Jurisdiction	QLD	1.00 (reference)	1.00 (reference)	1.00 (reference)	1.00 (reference)	1.00 (reference)	1.00 (reference)
	SA/WA	1.83 (0.70–4.78)	1.62 (0.60–4.34)	0.16** (0.08–0.34)	2.42 (0.72–8.13)	1.44 (0.71–2.92)	0.67 (0.29–1.55)
	NT	4.99** (2.93–8.51)	9.73** (5.68–16.67)	2.45** (1.70–3.54)	4.98** (2.55–9.73)	1.67** (1.14–2.46)	0.91 (0.58–1.43)
CQI experience	Baseline audit	1.00 (reference)	1.00 (reference)	1.00 (reference)	1.00 (reference)	1.00 (reference)	1.00 (reference)
	1–2 follow-up audits	3.15** (1.38–7.20)	2.18 (0.82–5.77)	2.51** (1.32–4.76)	3.73** (1.45–9.58)	2.06** (1.22–3.47)	1.60 (0.86–2.96)
	≥3 follow-up audits	1.26 (0.57–2.77)	1.15 (0.45–2.94)	1.70 (0.92–3.14)	1.03 (0.42–2.56)	1.20 (0.73–1.98)	1.04 (0.57–1.89)
Governance	Community-controlled	1.00 (reference)	1.00 (reference)	1.00 (reference)	1.00 (reference)	1.00 (reference)	1.00 (reference)
	Government	0.93 (0.42–2.10)	0.87 (0.35–2.17)	1.35 (0.72–2.50)	1.30 (0.51–3.34)	0.90 (0.54–1.51)	1.30 (0.73–2.31)
Location	Urban	1.00 (reference)	1.00 (reference)	1.00 (reference)	1.00 (reference)	1.00 (reference)	1.00 (reference)
	Regional	0.24* (0.06–0.96)	1.15 (0.23–5.74)	0.37* (0.15–0.92)	0.18* (0.03–0.91)	0.33* (0.14–0.80)	1.27 (0.42–3.77)
	Remote	0.89 (0.30–2.61)	2.97 (0.84–10.54)	3.30** (1.64–6.64)	0.51 (0.14–1.81)	0.63 (0.32–1.25)	1.75 (0.74–4.14)
Population size	<500	1.00 (reference)	1.00 (reference)	1.00 (reference)	1.00 (reference)	1.00 (reference)	1.00 (reference)
	500–1,000	0.36** (0.17–0.74)	0.33** (0.14–0.74)	0.48* (0.28–0.85)	0.33** (0.14–0.77)	0.57* (0.36–0.92)	1.30 (0.75–2.25)
	>1,000	0.37** (0.19–0.70)	0.28** (0.13–0.58)	0.46** (0.28–0.75)	0.32** (0.15–0.67)	0.54** (0.36–0.83)	0.69 (0.41–1.14)
**Client level characteristics**							
Age group	15–24 years	1.00 (reference)	1.00 (reference)	1.00 (reference)	1.00 (reference)	1.00 (reference)	1.00 (reference)
	25–34 years	1.29** (1.07–1.57)	1.41** (1.15–1.72)	1.28** (1.06–1.53)	1.09 (0.89–1.33)	1.11 (0.93–1.33)	1.33* (1.00–1.75)
	35–44 years	0.95 (0.76–1.18)	1.31* (1.03–1.66)	0.63** (0.51–0.79)	1.01 (0.80–1.29)	1.00 (0.81–1.23)	1.71** (1.26–2.33)
	45–54 years	1.05 (0.82–1.34)	1.72** (1.31–2.25)	0.93 (0.73–1.18)	1.11 (0.85–1.45)	1.10 (0.88–1.38)	2.05** (1.48–2.85)
Gender	Male	1.00 (reference)	1.00 (reference)	1.00 (reference)	1.00 (reference)	1.00 (reference)	1.00 (reference)
	Female	1.70** (1.46–1.99)	1.69** (1.45–1.98)	0.86 (0.75–1.00)	1.14 (0.96–1.34)	1.44** (1.25–1.66)	1.10 (0.89–1.36)
Indigenous status	Non-indigenous	1.00 (reference)	1.00 (reference)	1.00 (reference)	1.00 (reference)	1.00 (reference)	1.00 (reference)
	Indigenous	3.61** (2.33–5.61)	3.78** (2.28–6.25)	2.15** (1.49–3.08)	4.28** (2.31–7.93)	1.84** (1.31–2.60)	0.81 (0.46–1.44)
	Not recorded	0.79 (0.35–1.78)	0.28 (0.06–1.34)	1.29 (0.70–2.37)	0.40 (0.13–1.25)	0.69 (0.38–1.27)	0.52 (0.16–1.68)
Time since last attendance	≥6 months	1.00 (reference)	1.00 (reference)	1.00 (reference)	1.00 (reference)	1.00 (reference)	1.00 (reference)
	<6 months	2.94** (2.42–3.58)	2.58** (2.07–3.22)	1.96** (1.63–2.35)	2.14** (1.72–2.68)	2.18** (1.82–2.60)	1.15 (0.86–1.54)

**Table 4:** Adjusted multilevel logistic regression analysis of health center and client level factors on a composite indicator of guideline-scheduled preventive service items recommended for well adults (*N* = 3,623 clients, 101 health centers) (*p < 0.05; **p < 0.01) (see Table 1 for service items included in this indicator).

		Model A	Model B	Model C
		Odds ratio (95% confidence interval)		
**Fixed effects**				
**Health center level characteristics**				
Jurisdiction	QLD		1.00 (reference)	1.00 (reference)
	SA/WA		1.28 (0.49–3.32)	1.22 (0.47–3.13)
	NT		5.71** (3.60–9.06)	4.35** (2.74–6.90)
CQI experience	Baseline audit		1.00 (reference)	1.00 (reference)
	1–2 follow-up audits		3.68** (1.96–6.89)	3.68** (1.98–6.87)
	≥3 follow-up audits		1.47 (0.81–2.68)	1.54 (0.85–2.78)
Governance	Community-controlled		1.00 (reference)	1.00 (reference)
	Government		0.82 (0.43–1.56)	0.74 (0.39–1.39)
Location	Urban		1.00 (reference)	1.00 (reference)
	Regional		0.24** (0.09–0.64)	0.21** (0.08–0.58)
	Remote		0.30* (0.12–0.75)	0.26** (0.10–0.65)
Population size	<500		1.00 (reference)	1.00 (reference)
	500–1,000		0.57* (0.33–0.99)	0.50* (0.29–0.87)
	>1,000		0.36** (0.21–0.61)	0.38** (0.23–0.64)
**Client level characteristics**				
Age group	15–24 years			1.00 (reference)
	25–34 years			1.31** (1.07–1.59)
	35–44 years			0.96 (0.76–1.20)
	45–54 years			1.06 (0.82–1.37)
Gender	Male			1.00 (reference)
	Female			1.59** (1.35–1.87)
Indigenous status	Non-Indigenous			1.00 (reference)
	Indigenous			3.67** (2.37–5.68)
	Not recorded			1.07 (0.48–2.40)
Time since last attendance	≥6 months			1.00 (reference)
	<6 months			2.80** (2.29 –3.41)
**Random effects**				
	Health center level residual variance	2.13 (1.51–3.00)	0.85 (0.58–1.24)	0.82 (0.55–1.21)
	MOR (health center)	4.02	2.41	2.37
	PCV compared to Model A (health center)		60.09%	61.50%
	Client level residual variance	0.97 (0.72–1.30)	1.24 (0.34–4.56)	0.18 (0.05–0.68)

*Median odds ratio (MOR): odds of receiving above median service delivery if client was to change health center or jurisdiction; proportional change in variance (PCV): per cent variation explained in odds for better health care delivery by introduction of health center or client level factors*.

The PCV in Model B for each mode of care (additional files 1–5) ranged between 14 and 67% for different models of care with follow-up being the lowest and basic measurements being the highest. Health center factors associated with higher delivery were similar across modes of care (Table 5). There was some variation across jurisdictions with NT health centers significantly associated with higher delivery for all modes of care except for lifestyle risk factors and follow-up. The pattern of client level effects was similar across modes of care except for gender, where females were more likely to receive basic measurements and assessment and intervention for lifestyle risk factors but less likely to have laboratory and imaging investigations. There was some variation in the effect of age group.

**Table 5:** Adjusted multilevel logistic regression analyses of health center and client level factors on delivery of guideline-scheduled service items by mode of care showing only significant associations (see additional files for full model outputs) (*p < 0.05; **p < 0.01).

		**Basic measurements**	**Laboratory and imaging investigations**	**Eye, ear and oral checks**	**Assessment and brief intervention for lifestyle risk factors**	**Follow-up of abnormal findings**
	
	No. of client records	3,623	3,623	3,623	3,623	1,905
	No. of health centers	101	101	101	101	101

		Odds ratio (95% confidence interval)				
**Fixed effects**						
**Health center level characteristics**						
Jurisdiction	QLD	1.00 (reference)	1.00 (reference)	1.00 (reference)	1.00 (reference)	1.00 (reference)
	SA/WA		0.18** (0.09–0.35)			
	NT	9.12** (5.62–14.81)	1.83** (1.36–2.46)	4.98** (2.82–8.80)		
CQI experience	Baseline audit	1.00 (reference)	1.00 (reference)	1.00 (reference)	1.00 (reference)	1.00 (reference)
	1–2 follow-up audits	2.96** (1.56–5.64)	1.82** (1.22–2.73)	4.95** (2.31–10.61)	2.05** (1.26–3.34)	
	≥3 follow-up audits					
Governance	Community-controlled	1.00 (reference)	1.00 (reference)	1.00 (reference)	1.00 (reference)	1.00 (reference)
	Government		0.65* (0.43–0.99)			
Location	Urban	1.00 (reference)	1.00 (reference)	1.00 (reference)	1.00 (reference)	1.00 (reference)
	Regional		0.24** (0.12–0.48)	0.16** (0.05–0.56)	0.27** (0.12–0.58)	
	Remote			0.13** (0.04–0.40)	0.31** (0.15–0.65)	
Population size	<500	1.00 (reference)	1.00 (reference)	1.00 (reference)	1.00 (reference)	1.00 (reference)
	500–1,000	0.45** (0.26–0.80)			0.63* (0.41–0.97)	
	>1,000	0.32** (0.18–0.55)	0.60** (0.43–0.84)	0.33** (0.17–0.63)	0.50** (0.33–0.76)	
**Client level characteristics**						
Age group	15–24 years	1.00 (reference)	1.00 (reference)	1.00 (reference)	1.00 (reference)	1.00 (reference)
	25–34 years	1.40** (1.14–1.73)	1.27* (1.05–1.53)			1.32* (1.00–1.75)
	35–44 years	1.36* (1.06–1.73)	0.63** (0.51–0.78)			1.73** (1.27–2.36)
	45–54 years	1.82** (1.38–2.39)				2.07** (1.49–2.88)
Gender	Male	1.00 (reference)	1.00 (reference)	1.00 (reference)	1.00 (reference)	1.00 (reference)
	Female	1.39** (1.18–1.65)	0.80** (0.69–0.93)		1.37** (1.18–1.59)	
Indigenous status	Non-Indigenous	1.00 (reference)	1.00 (reference)	1.00 (reference)	1.00 (reference)	1.00 (reference)
	Indigenous	3.84** (2.35–6.26)	2.08** (1.45–2.97)	4.18** (2.29–7.63)	1.90** (1.34–2.69)	
	Not recorded					
Time since last attendance	≥6 months	1.00 (reference)	1.00 (reference)	1.00 (reference)	1.00 (reference)	1.00 (reference)
	<6 months	2.36** (1.89–2.95)	2.00** (1.67–2.41)	2.09** (1.67–2.62)	2.07** (1.73–2.49)	
**Random effects**						
Health center level residual variance		0.86 (0.58–1.26)	0.27 (0.17–0.23)	1.34 (0.92–1.95)	0.47 (0.32–0.70)	0.74 (0.47–1.17)
Client level residual variance		0.03 (0.01–0.12)	0.47 (0.19–1.16)	0.13 (0.02–0.68)	0.85 (0.30–2.45)	0.21 (0.05–0.94)

## Discussion

Australia has lacked high quality systematically collected information on the quality of preventive care delivered in general practice and other primary health care settings. This study examining the most comprehensive dataset of its kind currently available in Australia provides the most extensive snapshot of delivery of preventive care to Indigenous people across Australia to date. We discuss the key findings around three themes: (a) Variation between aspects of care and key opportunities for improvement; (b) follow-up of identified risk factors and abnormal clinical and laboratory findings; and (c) variation between health centers. Key considerations for policy, practice, and research arising from these findings are outlined in Table 6.

**Table 6:** **Key findings and considerations for policy, practice, and research**.

Key findings	Considerations for policy, practice, and research
Variation between aspects of care and key opportunities for improvement	Use CQI processes to identify and address priority areas for improvement (15)
	Use strategies or design options at various system levels to enhance delivery of priority aspects of preventive care, with a focus on addressing specific barriers at the patient, health center, regional, and policy levels
	Evaluate and refine CQI processes and other strategies to maximize suitability and effectiveness in different contexts
	Possible areas for specific focus include
	− Review appointment systems, walk-in arrangements and work flow in clinics to maximize opportunities for health assessments and preventive care (30)
	− Design processes to enable completion of health assessments over successive visits
	− Allocate specific time for completion of health assessments (31)
	− Provide training on priority aspects of preventive care for individuals and teams (7)
	− Review and clarify roles and responsibilities of health teams with regard to health assessments and preventive care
	− Provide decision support for completion of all recommended preventive services (32)
	− Consider design of gender specific services to meet local needs, including development of gender specific health worker roles
	− Use outreach to workplaces and family or other groups when appropriate to deliver health assessments and enhance preventive care for priority hard-to-reach groups
	− Support research to identify and address specific barriers to preventive care
Low levels of follow-up of identified risk factors and abnormal clinical and laboratory findings	Use CQI processes to identify and address priority areas for improvement in follow-up care
	Use strategies or design options at various system levels to enhance follow-up, with a focus on addressing specific barriers at the patient, health center, regional and policy levels
	Possible areas for specific focus include
	− Development of incentives or removal of barriers at the policy level – for example increased financial incentives for effective follow-up, or reducing the number of recommended preventive services to focus effort on ensuring follow-up (31)
	− Consider incentives and barriers at health service, community and patient level, for example cost and availability of health services, and of transport (30, 31)
	− Encourage effective use of clinical information systems to enhance follow-up, including clear documentation of planning and delivery of follow-up care (31) and provision of appropriate decision support
	− Consider how development and implementation of models of patient-centered care could enhance follow-up care
	− Ensure individual staff and health teams understand the importance of follow-up care
	− Support research to identify and address specific barriers to follow-up
Variation between health centers	Use CQI processes to monitor and address variation between health centers/districts/regions in delivery of preventive care, with an emphasis on enhancing delivery in health centers/districts/regions at the lower end of the range
	Possible areas for specific focus in understanding and enhancing preventive care in health centers/districts/regions at the lower end of the range
	− Support research to understand barriers and development and implementation of strategies to address variation
	− Support effectiveness of information technology and sharing of clinical information, including developing staff capability and improving user friendliness of clinical information systems in these health centers/districts/regions
	− Implement appropriate redesign and re-allocation of resources – including but not restricted to staff resources such as Aboriginal Health Practitioners, allied health professionals
	− Consider how organizational management and culture could be developed to enhance service delivery in these health centers/districts/regions
	− Consider how structure, function, skills and knowledge base of health teams could be developed specifically to enhance service delivery in these health centers/districts/regions
	− Explore how challenges of staff recruitment and retention, and provision of expert and experienced decision support could be implemented specifically to enhance service delivery in these health centers/districts/regions
	− Enhance opportunities for high performing services to share their systems and approach to care with those services with less well developed care delivery

### Variation between Aspects of Care and Key Opportunities for Improvement

There is substantial variation in the delivery of recommended preventive services; from about 70 to 80% for most basic measurements to less than 20% for mammogram screening for breast cancer. While comparison with previously published research is limited by the comparability of data, the patterns of service delivery evident in our study are generally similar to those previously described, with generally good delivery of basic measurements and lower documented delivery of recommended laboratory investigations (5, 8, 20). While our analysis is consistent with previous findings of reasonable levels of assessment of lifestyle risk factors (5, 8, 20), we found higher levels of brief intervention to address these risk factors than previously reported, especially for alcohol and tobacco use.

Several service items including measurement of BMI and waist circumference stand out for being simple and quick to complete yet having low levels of delivery. This might reflect a lack of decision support within the clinic records, patient refusal or reluctance among some clinicians to engage clients in discussion of their weight. As the vast majority of clients present for reasons other than a preventive health assessment (8), the context of an acute presentation may detract from providing preventive care to people with no diagnosed chronic disease and isolated and non-urgent risk factors.

Low levels of documented delivery of some laboratory tests may relate to practical barriers to laboratory services in the remote locations in which most services included in this study are operating. It may also reflect poor integration of pathology results into electronic health records.

The low level of mammogram screening may be due to limited access to medical imaging services in remote areas and reflects a wider problem of access to specialized services for Indigenous Australians (30). Mammography screening for remote communities relies on either mobile screening units with a limited number of visits, or patient travel to larger centers which is not currently funded in the NT (33).

Our findings that female gender, being aged 25–34 years and recent health center attendance are associated with higher delivery of preventive care are consistent with previous studies (5, 7). That better preventive care delivery was significantly higher for females compared to males is likely to reflect a number of factors, including differences in health-seeking behaviors, proactive women’s health staff and that health centers tend to be predominantly female staffed so may be seen as “women’s places.” The relatively lower level of preventive care delivered to males represents a key opportunity for improvement.

### Follow-up of Risk Factors and Abnormal Findings

Documented evidence of follow-up of various identified risk factors and of abnormal clinical and laboratory findings was poor (notably follow-up for abnormal BP, BGL, and serum lipids) and arguably represents the most significant opportunity for improving early intervention for chronic diseases. Reasons for lack of follow-up are varied and are present at the patient, health center, and policy levels (31). Lack of knowledge of the reasons for follow-up among some patients and therefore low demand, as well as concerns regarding transport and the cost of follow-up services may contribute to low levels of follow-up (31). Follow-up is likely to be relatively more difficult for clients who attend the health center infrequently; more than one quarter of clients in our study had not attended in the previous 6 months and many Indigenous people have a high level of mobility.

### Variation between Health Centers

In contrast with previous findings that client level factors were responsible for the majority of inter-client variability in delivery (5), we found measured health center factors explained the majority of variation in delivery of preventive care. Urban location, smaller service population, and location in the NT were associated with higher quality care.

Our findings support a previous study showing that a smaller health center service population is associated with greater adherence to best practice guidelines. However, in contrast to another finding of that study that committee or board operated health centers performed better than their government run counterparts we found no difference in outcomes between the two (5). The association of health center location in the NT may reflect greater investment in PHC, a longer and stronger history of engagement in CQI, partnership between ACCHS and government operated services, support for guideline implementation, and possibly greater commitment to information management compared to other jurisdictions.

In contrast to previous research, we found that urban location was associated with better delivery compared to regional or remote (5). This finding in particular should be interpreted with caution given the relatively small number and selected nature of urban health centers included in our study. Excluding one especially high performing urban health center from our analysis caused the effect of remote location compared to urban location to be insignificant for overall delivery, although regional location was still significantly associated with poorer delivery. If this effect does hold for the wider population of health centers, it may in part reflect greater access to referral services in urban locations as remote practitioners may be reasonably reluctant to carry out services such as visual acuity testing and oral health checks where there are more limited options for referral for treatment. It may also reflect resourcing and funding advantages in urban areas or a strategic decision among larger urban health centers to invest in Medicare funded health assessments as an opportunity to increase service remuneration. There has been wide variation in uptake of adult health assessments between health centers (6, 34), with substantial increases in urban and regional locations in the last few years and relatively little change in remote locations (34).

There are various factors that influence the effectiveness of PHC centers, which can be complex and difficult to measure, and may account for the unexplained variation between health centers (35). These include community linkages, organizational culture, effectiveness of team structure and function, degree of staff turnover, availability of Aboriginal Health Practitioners, allied health professionals and other resources, and use of information technology systems for recall and reminders (27, 35, 36).

Limitations of this study mostly relate to the generalizability of findings. Health centers participated on a voluntary basis were not randomly selected, therefore, our data may not be representative of all health centers in each jurisdiction. Furthermore, data refer only to those people who have attended participating health centers within the last 24 months and therefore do not provide reliable population estimates. The age and gender stratified samples are designed to facilitate comparison between communities and over time. Estimates based on this sampling approach may differ from those based on unstratified samples.

It must be emphasized that our data are based on recorded delivery, which generally underestimate actual service delivery (37). However, under-recording is problematic in team based care and in areas of workforce turnover as well as potentially resulting in unnecessary servicing, so accurate recording is important for improving quality of care (5). Assumptions made regarding prevalence of sexual activity and trachoma are likely to have led to underestimation of actual levels of delivery in relation to best practice guidelines while those concerning lipid testing and mammography may have led to some over- or underestimation.

## Conclusion

We assessed the delivery of recommended preventive health care for clients of Indigenous health centers. Wide variation in delivery between service items, low levels of documented follow-up for abnormal clinical findings, and the importance of health center factors in determining adherence to best practice guidelines provide valuable insights for improving quality of preventive care for Indigenous Australians. Improvement may be achieved by addressing physical, social, and cultural barriers to accessing preventive care, and by strengthening systems for follow-up and completion of preventive health assessments. Further clarification of the impact of health center factors such as resourcing, team structure and function, and use of clinical information systems will give better insight into possible improvements. Addressing identified gaps in preventive screening according to best practice and prioritizing interventions to address these can be implemented through CQI approaches that engage stakeholders at multiple levels (15, 38).

## Author Contributions

CB designed and conducted the analysis, and drafted the manuscript under the supervision of VM who has lead responsibility for ABCD NRP data management and analysis. All other authors contributed to design of audit tools and facilitation of quality improvement processes from which the data used in this study arise, and contributed to data interpretation and refinement of the manuscript.

## Conflict of Interest Statement

The authors declare that the above research was conducted in the absence of any commercial or financial relationships that could be construed as a potential conflict of interest.

## Footnotes

^1^In the interest of brevity Aboriginal and Torres Strait Islander people are hereafter respectfully referred to as “Indigenous.”
